# Do Transgender and Gender Diverse Individuals Receive Adequate Gynecologic Care? An Analysis of a Rural Academic Center

**DOI:** 10.1089/trgh.2019.0037

**Published:** 2020-03-16

**Authors:** Talia Stewart, Y. Angie Lee, Ella A. Damiano

**Affiliations:** ^1^ Geisel School of Medicine at Dartmouth, Hanover, New Hampshire.; ^2^ Department of Obstetrics and Gynecology, Dartmouth-Hitchcock Medical Center, Lebanon, New Hampshire.

**Keywords:** transgender, gender nonbinary, gender nonconforming, gender diverse, gynecologic care, rural

## Abstract

**Purpose:** The transgender population faces disparities accessing gynecologic health care services, especially in rural settings. There is limited knowledge among medical providers regarding transgender-specific gynecologic care.

**Methods:** A retrospective chart review of 255 transgender and gender diverse patients at a rural, academic center and associated ambulatory clinics was performed. Demographics, insurance status, and utilization rates of screening services, including cervical cancer, breast cancer, human papillomavirus (HPV) vaccination status, and contraceptive status, were analyzed using descriptive statistics. These rates were compared with national rates of cisgender individuals. Chi-square tests were performed to assess the association of insurance status with receipt of services.

**Results:** Prevalence of HPV vaccination was lowest among transgender men (20%) compared with transgender women (60%) and gender nonbinary/nonconfirming and gender diverse individuals (60%), *p*<0.001. Our cohort was significantly less likely to receive Papanicolaou smears (51% vs. 81%, *p*<0.05) and contraception (48% vs. 65%, *p*<0.05) than cisgender individuals. Around 18% of transgender women had a documented pelvic examination in the past year. There was no significant difference in utilization rates based on insurance status.

**Conclusion:** In our rural setting, there is lower utilization of gynecologic services among transgender and gender diverse individuals. Although participants in our study had high rates of access to insurance and health care providers, they still had lower rates of gynecologic screening and prevention services. To address these disparities, we advocate for developing transgender-specific gynecologic health maintenance guidelines, robust provider education, and an inclusive electronic medical record to ensure appropriate gynecologic health screening.

## Introduction

The transgender population is one of the most medically underserved populations and faces significant disparities accessing gynecologic health care services.^1,2^ Transgender men have lower rates of cervical cancer screening and Papanicolaou (Pap) tests, and one study documented that transgender male patients had 37% lower odds of being up to date on Pap tests compared to cisgender women.^3,4^

These health disparities also vary geographically. Research shows that living in a rural setting can increase the likelihood of isolation and discrimination against the transgender population.^5^ However, much of the research on the transgender population has been primarily conducted in urban areas and is very limited within rural communities.^6^ There is also evidence that transgender individuals experience barriers to health care in the form of lacking equitable access to quality health insurance, are more likely to be uninsured, and have no usual source of health care when compared to the cisgender population.^7^

In addition, there is little research regarding the specific sexual and reproductive health needs of this population.^8^ Consequently, existing guidelines involving breast/chest health, cancer screening, and prevention have been adapted from guidelines for cisgender individuals.

Given the current gaps in the literature, the objective of our study is to compare utilization rates of gynecologic screening services by transgender individuals in a rural setting, compared to the national utilization rates among cisgender individuals. We sought to determine if utilization rates differed by insurance type or gender identity to explore how this impacts access to health care.

## Methods

A retrospective chart review of participants presenting to Dartmouth Hitchcock Medical Center (DHMC), a 396-bed rural academic center located in Lebanon, New Hampshire (NH), and its associated ambulatory community clinics located throughout NH, was conducted. Participants were identified using the electronic medical record (EMR; Epic Systems Corporation) with the following inclusion criteria: age >18, receiving primary care with a Dartmouth Hitchcock affiliated provider, and not identifying as cisgender. Participants were identified either by “problem list” entries: “transgender,” “gender nonconforming (GNC),” “gender nonbinary (GNB),” “genderqueer,” “other gender identity,” “gender dysphoria (GD),” and “gender identity disorder (GID),” or provider entered *International Statistical Classification of Diseases* (ICD) and Related Health Problems^9^ diagnostic codes F64 and Z87.890. Chart review was performed on all included participants to determine participant-reported gender identity. For the purposes of this study, individuals were categorized into the following: gender diverse (including GID and GD), transgender women, transgender men, or GNB/GNC/genderqueer. Data were generated from encounters recorded from January 2015 to December 2018. Each medical recorded was reviewed for demographic and outcome measures.

Primary outcomes/interventions included cervical cancer (Pap smear) and breast cancer (mammogram) screening, human papillomavirus (HPV) vaccination, and contraceptive status. Screening and eligibility guidelines (Table 2) were obtained from the Guidelines for the Primary and Gender-Affirming Care of Transgender and Gender Nonbinary People, at the University of California, San Francisco,^10^ United States Preventive Services Task Force (USPSTF),^11^ American Society for Colposcopy and Cervical Pathology (ASCCP),^12^ Advisory Committee on Immunization Practices (ACIP),^13^ Food and Drug Administration (FDA),^14^ and The American College of Obstetricians and Gynecologists (ACOG).^15^ Chart review was performed for each participant to determine eligibility for screening, vaccination, or contraception. If eligible, the participant's medical record was reviewed to determine if the intervention was received. After reviewing the medical record, if it was determined that the participant was eligible for an intervention, yet did not receive the intervention, documents scanned into the record from outside facilities were reviewed. If the participant reported receiving screening, vaccination, or contraception at an outside institution, but these records were not scanned into the record, this was recorded as “intervention completed.” If it was still unclear whether the participant received the intervention after reviewing scanned documents and provider records, this was recorded as noncompliant. Participants not meeting eligibility criteria, but still receiving the intervention due to other indications, were classified as “screening not indicated.” For example, a 32-year-old individual, receiving a diagnostic mammogram for a suspicious lump, was classified as “breast cancer screening not indicated.”

**Table 1.** Baseline Sociodemographic Characteristics

Demographic category	Total (N*=255), *n (%)
Age	
18–24	103 (40)
25–35	81 (32)
36–45	33 (13)
>46	38 (15)
Sex assigned at birth	
Female	159 (62)
Male	96 (38)
Gender identity	
Transgender woman	87 (34)
Transgender man	146 (57)
GNB/GNC/Genderqueer	11 (4)
Gender diverse	11 (4)
GNB/GNC/Genderqueer/Gender diverse (*N*=22)	
AFAB	13 (59)
AMAB	9 (41)
Self-declared ethnicity	
Hispanic/Latino	9 (4)
Non-Hispanic/Latino	236 (93)
Declines to list/UNK	10 (4)
Self-declared race/Color	
White	236 (93)
American Indian/Alaska Native	4 (2)
Black or African American	1 (0.4)
Multiracial	3 (1)
Declines to list/UNK	11 (4)
Employment status	
Self-employed	10 (4)
Employed FT	91 (36)
Employed PT	22 (9)
Student FT	58 (23)
Retired	7 (3)
Not employed	53 (21)
Disabled	9 (4)
Declines to list/UNK	5 (2)
Insurance type/status	
Private only	142 (56)
Medicare or Medicare plus private	29 (1)
Medicaid or Medicare plus Medicaid	64 (25)
Uninsured	7 (3)
UNK	13 (5)
Provider type	
Pediatrician	32 (13)
Adult	219 (86)
Endocrinologist	4 (2)

FT, full time; GNB, gender nonbinary; GNC, gender nonconforming; PT, part time; UNK, unknown.

**Table 2.** Guidelines and Exclusion Criteria

Service	Guidelines	Excluded from sub-analysis
Cervical cancer screening	For transgender men, cervical cancer screening follows recommendations for cisgender women.^10^ The 2018 USPSTF guidelines for average-risk women include^11^ the following: • <21 years—no screening regardless of age at sexual debut • 21–29 years—cervical cytology alone every 3 years • 30–65 years—cervical cytology alone every 3 years OR HPV alone every 5 years OR cytology with HPV (co-testing) every 5 years Colposcopy indicated in accordance with ACOG guidelines on abnormal cervical cancer result follow-up testing.^12^	• Previous total hysterectomy (i.e., no cervix) • <21 years old • Those not meeting screening guidelines, but received pap testing for other indications
HPV vaccination	Initial guidelines (May 18, 2006) • AFAB born May 20, 1979, to May 19, 1997 (individuals in this age range would have been eligible for vaccination when initial guidelines were released on May 18, 2006).^13^ Recent guidelines (October 2018) • AFAB and AMAB, aged 9–45 were years eligible for vaccination.^14^	Per early guidelines (May 18, 2006) • AMAB • Born before May 20, 1979, or after May 19, 1997 Updated guidelines (October 2018) • >45 years old
Breast cancer screening	For transgender women who are 50 years old AND 5–10 year history of feminizing hormone use screening mammography is recommended every 2 years.^10^ Transgender men who have NOT undergone bilateral mastectomy, should follow guidelines of cisgender women.^10^ ACOG guidelines^15^ for average-risk women include the following: • Starting at 40 years, screening mammogram every 1–2 years • If screening has not commenced by age 40, begin screening no later than 50 years • Continue screening until at least 75 years.	• Those receiving diagnostic mammograms • Previous bilateral mastectomy
Contraception	Transgender men with the potential for pregnancy should be offered all forms of contraception offered to cisgender women.^10^	• AMAB • Post-menopausal • >49 years old v Partner AFAB

ACOG, The American College of Obstetricians and Gynecologists; AFAB, assigned female at birth; AMAB, assigned male at birth; HPV, human papillomavirus; USPSTF, United States Preventive Services Task Force.

All individuals >21 years of age, with a cervix were considered eligible for cervical cancer screening. To determine if the participant had a cervix, provider documentation and surgical history were reviewed. Chart review was performed according to Table 2 guidelines. Anal Pap testing was not evaluated in this study due to limited recording of this information in the participant's chart. For individuals with an abnormal result, further chart review was performed to determine if a follow-up colposcopy was performed. The charts of transgender women with a surgical history of vaginoplasty or penile inversion were reviewed, and documentation of Pap testing or pelvic exam was extracted. Due to lack of guidelines regarding Pap testing in transgender women, these individuals were not included in the final analysis.

For those eligible to receive the HPV vaccination, receiving at least one dose was considered “vaccine provided,” given data supporting one dose providing similar protection as three doses.^16^ Table 2 documents criteria used when performing chart review. HPV vaccination eligibility was categorized as both “new guidelines 2018” based on guidelines released in October 2018 and “original guidelines 2006” based on initial guidelines released in May 2006. This was done since 2018 guidelines were released less than a year before the start of this study and it was likely that participants had not yet presented to their provider's office to be offered this intervention.

Table 2 outlines mammography screening guidelines used during chart review. To determine length of time on hormones, chart review of provider documentation was performed. For eligible transgender women, screening within the past 2 years was considered “completed.” Eligible transgender men were considered noncompliant with screening if they were age 50–75 and had not undergone screening within the past year.

To study contraception and compare to a national cohort, categories of contraceptive status indicated by the Center of Disease Control (CDC)^17^ report (Table 3) were used. Chart review was performed for individuals 18–49 years of age (similar to the CDC sample age range 15–49), assigned female at birth (AFAB), and reporting sexual partners assigned male at birth. Those AFAB with unknown partner gender were also included. Contraceptive status was determined by chart review of provider documentation, current medication lists, and surgical history. Individuals not on contraception and not currently sexually active at the time of chart review were classified under “not using contraception—no intercourse in 3 months before interview.” Individuals who were currently sexually active and did not use contraception despite provider education on contraception, were categorized as “not using contraception-had intercourse in 3 months before interview.” Individuals who were not on contraception, and chart review could not verify that the provider discussed contraception or chart review indicated provider miseducation on contraception (suggesting testosterone therapy as appropriate contraception), were categorized as “not using contraception-no contraceptive counseling by provider.” Those who underwent sterilization themselves were compared to the CDC group “female sterilization” and those who had a partner undergoing sterilization were compared to the CDC group “male sterilization.” In accordance with the CDC report, when multiple methods of contraception were used, participants were classified according to the most effective method.

**Table 3.** Comparison of Utilization Rates in Our Sample Versus National Sample

Type of intervention	Our cohort	National cisgender	p
Cervical cancer	51% screened	81%^18^ screened	<0.05
HPV vaccination	46% (2006 guidelines) receiving vaccination	51.5%^19^ receiving vaccination	0.31
Breast cancer screening	53% screened	71.6% (50–74 years old)^18^ screened	0.88
Contraceptive status^17^	47.7% using contraception	64.9% using contraception	<0.05
Not using contraception, % (*n*)	52.3 (35)	35.1	—
Never had intercourse	4.5 (3)	10.2	
No intercourse in 3 months before interview	23.9 (16)	6.8	
Had intercourse in 3 months before interview	6.0 (4)	7.9	
No contraceptive counseling by provider	17.9 (12)	—	
Other^a^	—	10.2	
Using contraception, % (*n*)	47.7 (32)	64.9	—
Female sterilization (OR “Self sterilization”)	3.0 (2)	18.6	
Male sterilization (OR “Partner sterilization”)	—	5.9	
Oral contraceptive pill^b^	11.9 (8)	12.6	
Long-acting reversible contraception (IUD, implant)	13.4 (9)	10.3	
3-month injectable (Depo-Provera)	4.5 (3)	2.1	
Contraceptive ring or patch	1.5 (1)	1.2	
Diaphragm	—	-	
Condom	13.4 (9)	8.7	
Other^c^	—	5.6	

^a^ Includes surgically sterile—female (noncontraceptive), nonsurgically sterile—female or male, pregnant or post-partum, seeking pregnancy.

^b^ Includes two participants on progesterone-only pills and six participants on estrogen-containing pills.

^c^ Includes periodic abstinence—calendar rhythm or natural family planning, withdrawal other methods (includes emergency contraception, female condom, foam, cervical cap, sponge, suppository, and jelly, as well as “other methods”).

Subanalyses were performed to evaluate the association of utilization rates based on type of insurance, and rates in our sample were compared to national utilization rates of services in cisgender individuals. GNB/GNC/Genderqueer were combined with gender diverse for gender identity analyses, due to the small sample sizes in these groups.

The study was reviewed and approved by the Dartmouth-Hitchcock Medical Center IRB (study #31368). Descriptive statistics, such as frequencies and percentages, were used to describe categorical, continuous, and binary variables. To assess raw associations of primary outcomes with insurance status, and to determine utilization based on gender identity, a Pearson chi-square test (or Fisher's exact test if necessary) was used. A two-sample proportion chi-square test was used for subanalyses. All statistical analyses were performed using StataSE with a defined significance of *p*<0.05.

## Results

**FIG. 1.**
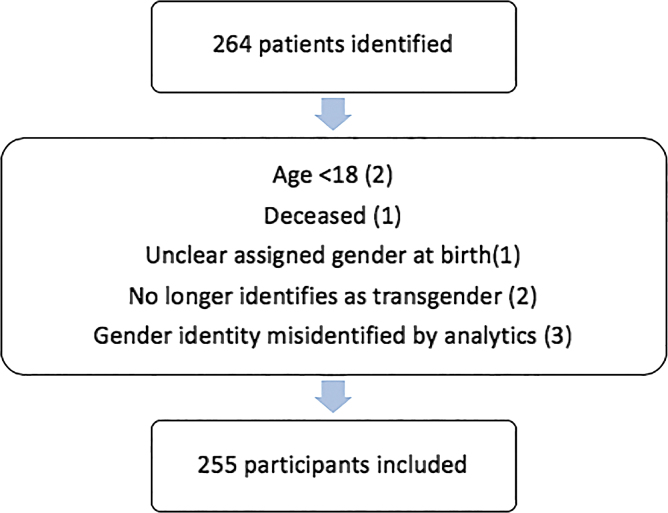
Exclusion criteria.

Patient demographic characteristics are provided in Table 1. Two hundred sixty-four participant charts were identified, and after excluding 9 participants, 255 (97%) participant charts were analyzed (Fig. 1). The mean age of the sample was 31 years. Sixty-two percent of participants were AFAB. The majority of our cohort identify as white and non-Latino (93%), consistent with the majority of the population in NH. Fifty-seven percent of participants identify as transgender men, 34% as transgender women, 4% as GNB/GNC/Genderqueer, and 4% as gender diverse. Of the GNB/GNC/Genderqueer/gender diverse cohort, 13 were AFAB and 9 were assigned male at birth. Thirty-six percent of participants were employed full time. Most were insured with commercial/private insurance (56%). The majority of participants (86%) had a provider who was an adult/family practice provider.

Out of 255 participants, 42% (*N*=108) were eligible for cervical cancer screening, based on criteria in Table 2. Fifty-one percent (*N*=55) received appropriate screening, while 11% (*N*=12) declined to be screened, deferred screening or were still considering whether or not they wanted to be screened. There was no statistically significant difference between prevalence of cervical cancer screening between eligible transgender men compared to GNB/GNC/Genderqueer/gender diverse individuals AFAB (*p*=0.46). Five individuals had an abnormal pap smear that warranted further follow-up, with 80% (*N*=4) receiving follow-up colposcopy. Eleven transgender women had a surgical history of vaginoplasty, or penile inversion. Eighteen percent (*N*=2) had a documented pelvic examination in the past year. None had a documented Pap test.

According to 2006 guidelines, 36% (*N*=93) of the sample was eligible to receive the HPV vaccination, with 46% (*N*=43) receiving the vaccination. According to 2018 guidelines, 84% (*N*=218) of the sample was eligible to receive the HPV vaccination, with 47% (*N*=102) receiving the vaccination. There was no statistically significant difference between receiving at least one dose of HPV vaccination according to 2006 guidelines when comparing transgender men, transgender women, and GNB/GNC/Genderqueer/gender diverse individuals (*p*=0.40). When using 2018 guidelines, there was a statistically significant difference, with 20% of transgender men, 60% of transgender women, and 60% of GNB/GNC/Genderqueer/gender diverse individuals receiving the vaccination (*p*<0.001).

Breast cancer screening was indicated for 7% (*N*=17) of the sample, based on criteria in Table 2. Fifty-three percent (*N*=9) received screening and 18% (*N*=3) declined screening. There was no statistically significant difference between prevalence of breast cancer screening between eligible transgender men, transgender women, and GNB/GNC/Genderqueer/gender diverse individuals (*p*=0.38).

Of our sample, 26% (*N*=67) was eligible to receive contraception. Table 3 shows contraceptive status of our cohort compared to CDC reported national data.^17^ Three participants currently using an estrogen containing oral contraceptive pill or estrogen-containing vaginal ring were also currently on testosterone hormone therapy. Eighteen percent (*N*=12) were not on contraception and there was no documentation of provider education about contraception. There was no statistically significant difference in contraception use when comparing eligible transgender men to GNB/GNC/Genderqueer/gender diverse individuals who were AFAB (*p*=0.46).

Table 3 compares utilization rates of our sample to national utilization rates of cisgender individuals. Our cohort was less likely to receive cervical cancer screening (51% vs. 81%,^18^ *p*<0.05), and less likely to be contracepted (48% vs. 65%,^17^ *p*<0.05). Our cohort was also less likely to receive breast cancer screening (53% vs. 72%,^18^ *p*=0.88) and HPV vaccination (46% vs. 52%,^19^ *p*=0.40), although these results were not statistically significant.

Table 4 compares utilization based on insurance status. There was no statistically significant difference between prevalence of any health maintenance screening, receipt of HPV vaccination, and using contraception based on type of insurance.

**Table 4.** Screening, Vaccination Rates, and Contraceptive Status Based on Insurance Status, Subanalysis

Service	Total No. eligible	Private only (N*=142), *n (55.69%)		Medicare or Medicare plus private (N*=29), *n (11.37%)		Medicaid or Medicare dual eligible^a^ (N*=64), *n (25.10%)		Uninsured (N*=7), *n (2.75%)		Unknown (N*=13), *n (5.10%)		p
		No. eligible	% received	No. eligible	% received	No. eligible	% received	No. eligible	% received	No. eligible	% received	
Cervical cancer screening	108	67	54	6	33	24	63	6	33	5	0	0.11
2006 HPV vaccination guidelines	92	58	41	4	50	22	55	5	60	3	33	0.77
2018 HPV vaccination guidelines	218	127	53	16	25	57	40	6	67	12	33	0.11
Mammogram	17	7	71	5	60	3	0	—	—	2	50	0.06
Contraception use^b^	67	39	51	4	50	18	44	2	50	4	25	0.73

^a^ Dual eligible is defined as those eligible for Medicare and Medicaid benefits.

^b^ Percentage represents percentage of participants using contraception, where contraception includes sterilization of self or partner, oral contraceptive pill, long-acting reversible contraception (intrauterine decide, implant), 3-month injectable (Depo-Provera), contraceptive ring or patch, or condom.

## Discussion

The objective of this study was to compare utilization of gynecologic preventative services by transgender individuals living in a rural setting, to national utilization rates among cisgender individuals. We also sought to determine if utilization rates differed by insurance type or gender identity.

We found significantly lower rates of contraception use and cervical cancer screening in our population compared to national rates and no significant difference in utilization based on health insurance type. A recently published *JAMA* article analyzing 2014–2017 Behavioral Risk Factor Surveillance System data (BRFSS)^20^ reported 79.9% of transgender individuals are insured compared to 85.4% of cisgender respondents. Our population is unique, as 92% were insured and <3% were uninsured, suggesting that no matter how robust the insurance coverage, transgender and gender diverse individuals still face health inequities. For example, Table 5 shows a general trend toward lower utilization rates in our rural cohort compared to urban settings. Transgender individuals living in rural areas often experience increased stigmatization by health care providers,^21,22^ leading to avoidance of seeking health care services due to fear of discrimination.^23^

**Table 5.** Screening Rates in Urban Settings Compared to Our Rural Cohort

Service	Study authors (year)	Study type, location	Utilization rates in urban settings	Utilization rates in our study (rural setting)
Cervical cancer	Agénor et al. (2016)^36^	Survey, Greater Boston	77.1%	51%
	Cipres et al. (2016)^37^	Retrospective chart review, San Francisco, CA	69%	
	Peitzmeier et al. (2014)^4^	Retrospective chart review, Boston, MA	64.3%	
	Porsch et al. (2016)^38^	Internet-based survey, NYC	83%	
HPV vaccine	Gorbach et al. (2017)^39^	Survey, Chicago, IL and Los Angeles, CA	14%	(2006 guidelines) 46%
Breast cancer	Bazzi et al. (2015)^40^	Retrospective chart review, Massachusetts	50%—Transgender men 54.9%—Transgender women	53%
	Clavelle et al. (2015)^41^	Cross-sectional, retrospective review, Northeast	42%	
Contraception	Cipres et al. (2016)^37^	Retrospective chart review, San Francisco, CA	42% report no method of birth control	52.3% report no method of birth control

Surveys show that up to 70% of health care providers report unfamiliarity with screening recommendations for transgender individuals,^24^ which is, in part, due to lack of health maintenance guidelines specific to transgender patients. Moreover, this may lead to low-quality care and poor recommendations. For example, in our cohort, three transmasculine participants on testosterone were using estrogen-containing contraception. There are currently no contraindications to using estrogen-containing contraception in transmasculine individuals on testosterone hormone therapy, as previous studies show these individuals maintain blood estradiol levels within the expected range of transmasculine individuals using testosterone.^25,26^ Yet the literature recommends that transmasculine individuals using testosterone avoid estrogen-containing contraceptives as to not counteract the masculinizing effects of testosterone.^27^ The inconsistencies in the literature complicate provider counseling, underscoring the necessity for further research on the effects of combining estrogen contraceptives and testosterone therapy in transmasculine individuals.

Similarly, health maintenance screening in transfeminine individuals, status post-vaginoplasty, is another area of ambiguity. While these individuals are not at risk for cervical cancer, they are at risk for HPV and other sexually transmitted infections.^28^ In our cohort, few transgender women who underwent vaginoplasty had a documented pelvic examination over the past year and none had documented Pap testing. Review of provider notes revealed two cases of providers documenting, “Pap does not apply because no cervix present.” However, a study conducted in the Netherlands tested neovaginal swabs for HPV in transgender women and discovered that 20% of sexually active transgender women tested positive for high-risk HPV compared to zero percent of sexually inactive transgender women.^28^ It is imperative that formal guidelines also be established for HPV screening in transgender women who undergo neovaginal reconstruction.

Our study also highlights areas in which physicians provided erroneous recommendations to transgender and gender diverse patients. In two cases, providers documented counseling transmasculine patients that testosterone therapy alone provides adequate contraception, although previous reports^29^ have proved this to be false. In parallel, our cohort AFAB showed significantly lower cervical cancer screening rates, which may be due to a misconception among providers and patients that transgender men not engaging in penile-vaginal intercourse do not require regular screening.^30^ While transmission of HPV does most frequently occur with penetrative sexual intercourse, it can occur following nonpenetrative sexual activity,^31^ justifying established guidelines advocating for regular screening if the individual has a cervix, regardless of sexual partner or practices. Also of public health concern, while cervical cancer is the third most common cause of death among gynecologic cancers in the United States,^32^ it is also one of the most preventable since the formulation of the HPV vaccination. Our cohort transgender men also had significantly lower rate HPV vaccination when compared to other gender identities in our cohort, which may signal avoidance of gynecologic preventive services from a young age.

Addressing the topics of cervical cancer screening and contraception in transgender and gender diverse patients requires the most care and sensitivity by providers. Both Pap testing and an unplanned pregnancy can heighten feelings of GD and psychological discomfort among transgender men. Transgender men who had been pregnant after transitioning have cited feelings of post-partum depression, and increased dysphoria due to not passing as a male while pregnant.^29^ Similarly, Pap tests have been described as a “threat to gender identity,” and surveys show that gynecologic examinations may result in a conflict between self-perceptions and physical anatomy.^33^ Interestingly, patients who felt respected and supported by their provider reported fewer feelings of GD and instead experienced a sense of pride in taking care of their health, evidence that provider sensitivity and counseling are critical.^34^

Our study is not without limitations. Participants were included if they identified a primary care provider at DHMC. This introduced selection bias into our study, but was important to increase the likelihood of a complete medical record in our system. Limiting generalizability is that 93% of our cohort identified as white. Third, although our overall sample size was large, there were small sample sizes in many categories, and our results may not have been adequately powered to detect differences between insurance types. Fourth, our comparison studies for cisgender rates may not provide a matched cohort to our rural sample. Our institution is unique in providing a high density of gynecologic providers in a rural setting compared to other rural counties.^35^ Fifth, our HPV-related screening did not address anal pap smear screening—an area for future research. Last, even though an inclusion criterion was having a primary provider in our health system, it is possible that our participants received care at outside facilities and were misidentified in our analysis, leading to an underestimation of utilization rates.

In summary, our study demonstrated lower utilization rates of screening services among transgender and gender diverse individuals living in a rural setting, which was surprising given that our entire sample had a primary care provider, and a majority of our sample was insured. It is critical to address the stigma and discrimination against the transgender population in our health system, which occurs due to lack of education and a noninclusive EMR. Underscoring this point is the lack of standard, transgender and gender diverse specific guidelines available to inform the gynecologic health care needs of transgender individuals. Providers should advocate for more robust education on transgender-specific care, including curriculum changes in medical schools and residency programs. The EMR should be restructured and include less gender normative documentation. In the future, we plan on making this a longitudinal study by conducting further follow-up of the participants in this group to determine whether utilization rates improve after implementation of our transgender gynecology clinic.

## Abbreviations Used

ACIP: Advisory Committee on Immunization Practices
ACOG: The American College of Obstetricians and Gynecologists
AFAB: assigned female at birth
AMAB: assigned male at birth
ASCCP: American Society for Colposcopy and Cervical Pathology
BRFSS: Behavioral Risk Factor Surveillance System data
CDC: Center of Disease Control
DHMC: Dartmouth Hitchcock Medical Center
EMR: electronic medical record
FDA: Food and Drug Administration
FT: full time
GD: gender dysphoria
GID: gender identity disorder
GNB: gender nonbinary
GNC: gender nonconforming
HPV: human papillomavirus
ICD: *International Statistical Classification of Diseases*
NH: New Hampshire
Pap: Papanicolaou
PT: part time
UNK: unknown
USPSTF: United States Preventive Services Task Force
